# Chinese English as a Foreign Language Teachers’ Wellbeing and Motivation: The Role of Mindfulness

**DOI:** 10.3389/fpsyg.2022.906779

**Published:** 2022-05-19

**Authors:** Menglai Pan, Jieying Liu

**Affiliations:** ^1^School of International Studies, University of International Business and Economics, Beijing, China; ^2^Guangxi International Business Vocational College, Nanning, China

**Keywords:** wellbeing, English as a foreign language teachers, motivation, mindfulness, EFL teachers

## Abstract

Teaching is a career with great instances of anxiety and exhaustion in all stages of education with particular difficulties associated with the attribute of language instruction. The notion of motivation might be a significant fundamental mechanism since demotivated educators are distressed due to the anxious feature of the instructing career. Moreover, educators’ wellbeing has been demonstrated to have a pivotal function in the path of instruction and learners’ success. On the other hand, to mitigate both motivation and wellbeing among teachers, one of the mental traits in this filed, namely, mindfulness can be effective as it is a technique that link to positive effects when used as an administrative strategy for alleviating stress and concern that bring about motivation and wellbeing. As a result, the purpose of the study is to investigate the predictor role of mindfulness on teachers’ motivation and wellbeing. In this study, 577 teachers (235 males and 342 females) Chinese English as a foreign language (EFL) teachers at different colleges, universities, and institutes in 13 provinces among which Jiangsu and Zhejiang province accounted for 26.69%, while other provinces made up 65.86% and 2 municipalities directly under the central government (Beijing and Chongqing; 7.45%). were kindly accepted to participate in the present study, and they answered the three questionnaires, namely, motivation, mindfulness, and wellbeing. The results of the study through a linear regression analysis indicated that teachers’ mindfulness could significantly predict both teachers’ wellbeing and motivation. According to the results, some pedagogical suggestions for the policymakers, educator trainers, materials developers, and language educators are offered. Ultimately, guidance for further studies is proposed to L2 scholars who are interested.

## Introduction

Educators are crucial factors in the body of healthy communities; however, their career is famous for its stress and it seems to be worsening as time passes (McIntyre et al., 2017). Studies on foreign language educators are important since they are the guides for foreign language students and without their direction, numerous students would fall in the dark. Comprehending educators’ psychology is required since their psychologies and expert wellbeing have been demonstrated to be related to the standard of their instruction and learners’ presentation (Mercer, 2018). Educators with constructive mindsets are prone to take pleasure in their careers and portray more creativity and advanced instructing abilities (Mercer, 2018). Prosperous language learning largely relies on educators; therefore, it seems necessary to prioritize their wellbeing (Mercer et al., 2016). An active structure of PP is wellbeing which alludes to the manner people contemplate, understand, and condition at a predetermined time (Greenfield, 2015). The fostering construct is usually considered when speaking about wellbeing in PP (Kern et al., 2016). Research on educators’ wellbeing can assist with deciding how they can successfully attain their instructional objectives. Existing research proposes that educators’ wellbeing greatly influences how they act, how they are involved with their students in the class, and their determination to persist when encountering hardships (MacIntyre and Gregersen, 2012; MacIntyre et al., 2019). Essentially, their understanding of their sensations and wellbeing relies on their main capability to adjust their affections, supervise and assess their behavior, and choose proper education tactics. A large body of literature suggests that the understanding of wellbeing is a vital instrument for language educators (Day and Gu, 2014; Hiver and Dörnyei, 2017).

The pieces of literature emphasize the tension-stimulating nature of education jobs and several outcomes of apprehension, though there is a lack of sufficient knowledge of what makes teachers’ capability of fostering within the work area less complicated (Jennings and Greenberg, 2009). Based on such developments, learners, policymakers, and administrators have got higher engagement in the educators’ wellbeing (Collie et al., 2012). The definition of educators’ wellbeing, as a basic construct of constructive psychology, is the degree of educators’ judgment and salinification about their intellectual and bodily circumstances (Song and He, 2021). Based on the studies, educators’ wellbeing is a vital factor in their effectiveness, retention as well as their students’ wellbeing. Consequently, improving wellbeing among educators is beneficial for themselves and the institutes’ achievement (Giorgi et al., 2017). Moreover, Positive Psychology (PP) is associated with interpersonal dimensions of wellbeing, and interferences were designed which enhance kindness, appreciation, and pardoning (McCullough and Van Oyen Witvliet, 2005).

Besides, sustaining a great degree of motivation is regarded by numerous scholars and psychologists as a significant standard that educators must build (Gillet et al., 2012). Being cognizant of the significance of motivation in learning, Deci and Ryan (2016) have suggested SDT as a novel viewpoint for people’s motivation. Educator motivation is a complicated notion put into consideration within the past 10 years and affects learner motivation and function (Dörnyei and Ushioda, 2011). An educator with a good standard of motivation goes onboard and accomplishes his or her assignments with a feeling of enthusiasm and joy. The origin of a good standard of motivation comes from the inside (innate) without the need for any relational or intrapersonal enforcement (Neves de Jesus and Lens, 2005).

Mindfulness construct is a constructive psychological resource that is almost ignored. Particularly, a mindful individual has elevated cognizance of the status quo and offers central attention to dwelling in the present time (Brown et al., 2007). Mindfulness, the unbiased, current cognizance of experience, came into existence as a prosperous handling mechanism to lessen tension and enhance career and mental wellbeing (Roeser et al., 2012). Mindfulness practices are compliant with a large number of PP theories and practices. Positive Psychology is an extensive umbrella related to the scientific perception and improvement of what leads to proper living (Seligman, 2011; Wang et al., 2021). Many studies regarding PP have concentrated on the proof for the advantages of constructive affections and the manner of enhancing them (Lyubomirsky et al., 2005). Although interferences in PP regularly concentrate on specific results, a mindfulness method affects a range of such results. The definition of mindfulness is the condition of understanding and comprehending what a person is experiencing (Wang and Liu, 2016). Sometimes, it involves understanding that the individual manages such incidences and is not passive (Schoeberlein and Sheth, 2009). Recently, it became evident that by reducing stress levels and improving coping strategies, mindfulness improves academic delivery and exam presentation (Dundas et al., 2016). Intervention based on mindfulness is proved to help students in coping with apprehension and in improving their socio-emotional performance (O’Driscoll et al., 2018).

The literature indicates the methods to study educators’ wellbeing and motivation are various. For instance, a large body of studies has been conducted to make sure how educator motivation can be enhanced to build a better education and learning setting (Brown et al., 2002; Gordon, 2002). Alternatively, the success of mindfulness plans has been documented in new research portraying that school educators who took part in the mindfulness plans documented greatly enhanced wellbeing and diminished anxiety and worry (Gold et al., 2010). As much as the researcher knows, only a few studies were associated with wellbeing and motivation among EFL Chinese educators on the one hand and the mindfulness role as a method in such sphere on the other hand. Therefore, the present research investigates whether mindfulness contributes educators to having higher awareness about the current moment and interdependently increase their motivation quality and their wellbeing.

## Review of the Literature

### Teacher Motivation

Motivation offers the primary impetus in language education and after some time the justification for going after the extended and dull educational cycle (Dörnyei, 2005). Dörnyei maintained that motivation adds to the entire components associated with attaining information regarding a second language. Moreover, it is mentioned that motivation is related to the reason behind individuals’ particular decision-making, involvement in an assignment, and perseverance in following it and it controls the magnitude of strong points and individual involvement in studying a second language (Ushioda, 2008). Motivation is a personal contrast factor, which acts as a driving force or encouragement to take action or to do something. It is enthusiasm and resolution with a sort of thrill that results in persistence to attain higher heights, regardless of the path of their life (individual or expert), and is primarily related to the stimulation of objective-directed manners (Singh, 2011). Motivation is normally a primary element in language attainment of forming student manner. For example, Martinez et al. (2016) believed that there is a mutual connection between motivation and strategy practice and that motivation results in strategy practice and vice versa. The literature on motivation accentuates three classes; in other words, innate motivation to know, innate motivation to achieve, and innate motivation to go through provocation (Ryan and Deci, 2020). Therefore, based on the self-determination hypothesis, people with innate motivation have an intrinsic source of power and inherent knowledge, take pleasure in achieving an assignment, are involved in exercises that lead to the encounter of provoking feelings, and are keen on studying novel matters (Deci and Ryan, 2016).

However, extrinsic motivation is primarily related to the influential worth of the presentation of an exercise to achieve some distinct result (Ryan and Deci, 2020). It is concerned with a set of manners that act as a means to an end as opposed to the end itself. Similar to innate motivation, external motivation involves three kinds: extrinsic control, which is regulated through external prizes, reprimands, and limitations; introjected control, which deals with internalizing the significance of and motives behind specific actions; and specified control, which deals with selecting to specify the worth related to education (Komarraju et al., 2009). Motivation can be characterized as preparation and hope to participate in decent instruction. There are two various kinds of motivation: autonomous and regulated motivation. On the one hand, autonomous motivation refers to acting with a feeling of determination, encountering choices; on the other hand, regulated motivation alludes to acting with a feeling of compulsion (Fokkens-Bruinsma et al., 2018). The former is associated with greater degrees of mental wellbeing, more will and resolution, better intellectual abilities, greater degrees of career satisfaction, and institutional devotion (Fokkens-Bruinsma et al., 2018).

### Mindfulness

Mindfulness is popular in the field of constructive psychology which has vital advantages, namely, enhancing working memory, improving wellbeing, lowering tension, etc. (Brown and Ryan, 2003). It is believed that the source of mindfulness originates in the old traditions of Hindu and Buddhist. Mindfulness refers to having a perfect awareness of the moment and others that are conscious of their physical, psychological, and communal experiences along with their senses, feelings, psychological pictures, and ideas while admitting them all with no bias (Taylor et al., 2016). Mindfulness is described as “a mental state or quality that is raised by focusing on the goal, currently, and in an unbiased way to disclosing experience every moment” (Kabat-Zinn, 2003) and it is regarded as a procedure instead of a task product; instead of being static, it is dynamic because it concentrates on continued experiences in life. The consciousness of unbiased experiences in each moment regarding such experiences is a main notion within the mindfulness descriptions. Mindfulness consciousness is the current experiences and incidents that can be gained through paying attention (Brown and Ryan, 2003) and it is an uncomplicated activity, a method of exercising the capability to focus on what is taking place in the surroundings (Wax, 2016). As a complicated and distinctive construct, mindfulness is regarded a notion comprising multiple factors. It involves individual and career characteristics assisting educators to link with diverse characteristics of experiences in life (Yuan et al., 2020).

Mindfulness is about consciousness and admittance, as well as the capacity to adjust and redirect one’s concentration in a beneficial manner (Bishop et al., 2004). Mindfulness means self-adjusting the concentration to one’s present experience and a specific tendency to such experience which features inquisitiveness, accessibility, and admittance (Bishop et al., 2004). Mindfulness includes deliberately and continually focusing on ones’ continued experiences in terms of senses, cognition, and affective experience with no explanation or judgment regarding any section of such experience (Kabat-Zinn, 2003). The psychologist, Ellen Langer mainly introduced and presented the mindfulness notion, even though it has some origins in east philosophy. In their study, Leary and Tate (2007) also defined mindfulness as moment attention arguing that it helps us in many diverse methods, the most vital of which are as the following: (a) it lessens negative future and thoughts of the past, self-admiration and judging through lowering what they referred to as “self-chatting.” (b) It enables us to admire the existing condition without complying with it or denying it. In other words, it inspires tranquility by looking at the current condition through an objective view. (c) It can improve prosocial conduct and constructive feelings with the aid of improving extra effective interaction approaches with different people. (d) it refrains from the fatigue of self-adjusting power sources. Educators’ mindfulness assists with promoting the creation of constructive associations with learners, affecting their mental elements, and building their expert growth, which could result in learners’ greater success (Sharp and Jennings, 2016). Mindful exercises can assist students with conquering disturbances and maintaining their concentration on their environment. In the class setting, mindfulness assists educators with being conscious of their idle pedagogical competencies so they can recognize what takes place in the surrounding. Moreover, mindfulness exercises assist educators with improving their comprehension of the body (like exhaustion), mind (contemplating competence), and sense of anxiety (Bernay, 2014).

Mindfulness assists educators with being constructive and powerful in difficult circumstances, thereby resulting in students’ educational success (Jennings and Greenberg, 2009). Moreover, exercising mindfulness elevates educators’ expert presentation (Schussler et al., 2016). By practicing mindfulness and making it a noticeable habit, enduring benefits for learners and teachers can be guaranteed (Altan et al., 2019). Mindfulness exercises have been demonstrated as successful in psychological healthcare settings; for example, based on observational research, individuals performing ritual meditation practices for 8-weels documented fewer emotive anguish symptoms and more mindfulness and psychological wellbeing (Carmody and Baer, 2008).

### Wellbeing

Acton and Glasgow (2015) believe that educator wellbeing is described as “people’s experience of individual expert accomplishment, delight, determination and joy, built in a cooperative procedure with coworkers and learners” (Acton and Glasgow, 2015). Wellbeing takes various forms and smoothes regarding people, family, and social beliefs, valuations, culture, experiences, chances, and settings throughout time and alterations. We all want it, supported by constructive concepts, however, it is particular to each individual and gives people an experience of who they are which needs some respect (McCallum et al., 2017). According to Ryan and Deci (2020), wellbeing is described in two views, the hedonic and eudemonic kinds. The first view is merely pertaining to satisfaction and life leisure and defines wellbeing as gaining joy and avoiding pain. The most important goal of the hedonic method is increasing satisfaction, forming what is to be recognized as “subjective wellbeing” (Kim-Prieto et al., 2005). However, the second method of wellbeing is centered on self-actualizing and includes the characteristic of personal life and achievement of capabilities. This attitude advocates the basis for psychological wellbeing and uses such skills to make use of assets in a manner that can make life meaningful (Mercer and Gregersen, 2020). In fact, such essential methods show the theory of wellbeing that highlights a long-run vision on meaning and dedication to joy, rather than a short-term hedonistic perspective of joy and satisfaction. Meaning that after people encounter consistent constructive feelings, are involved in numerous areas in life, communicate with different people, and attain their goals, then they are fostering, or certainly going through high-quality wellbeing (Wang and Guan, 2020).

The Wellbeing concept includes having a good living and connects to the concept of life quality mentioned above. Indicated in the word “flourish,” an attitude on wellbeing consisting of both happiness and delight, as well as significant involvement and ethical value is indicated (Seligman, 2011). The wellbeing components are included within the framework of PERMA. Frameworks were employed both as a motive to practical measure and investigation approaches. For example, Seligman’s (2011) PERMA framework encompasses Positive emotions, Engagement, Relationships, Meaning, and Accomplishments. Engagement is commonly referred to as a type of flow or deep engagement which is initially meant to be motivating all through task fulfillment, target determination, watching, and achieving high wellbeing during life (Derakhshan, 2021). A constructive relationship means a feeling of being admitted, paid attention to, and reinforced in social terms, and enjoying one’s relationship in society. Social assistance is related to constructive results of mental and bodily wellbeing in addition to wellbeing in general terms (Greenier et al., 2021). Yang (2021) believes that meaning refers to the notion that people’s lives have perseverance and a means within the life route which pertains to being related to something larger and with positive sensations in various ranges of age. Achievement is generally connected to target determination, development, and gaining the potential to attain, and as a result, attempting for wellbeing (Fredrickson, 2001).

## Materials and Methods

### Participants

The participants of this study were 235 males and 342 females Chinese EFL teachers at different colleges, universities, and institutes in 13 provinces among which Jiangsu province and Zhejiang province accounted for 26.69%, while other provinces made up 65.86% and 2 municipalities directly under the central government (Beijing and Chongqing; 7.45%). Their age ranged from 20 to 62, with their age group 31–40 and 41–50 occupying 40.21 and 36.57%, respectively. Informed consent was given to all participants. All of the data collected were based on the respondents’ willingness.

### Instruments

The following instruments are used in the present study.

#### Mindfulness Questionnaire

Frank et al. (2016) built the Inventory of MTS-C mindfulness utilized in this research, which involves 14 inquiries equivalent to its first version in English. The scholar asked the subjects to read declarations that explained the precision of each declaration for them in the past month on the root of 1 (always false) to 5 (always true) Likert scale. In the current study, the scale’s estimated Cronbach’s alpha reliability coefficient was 0.720.

#### Teachers’ Motivation Questionnaire

A ten-item questionnaire was developed by Dweik and Awajan (2013) so that people could answer the inventory. Teachers need to give their motivation rates to motivational bases through a five-point Likert scale. In the present study, the scale’s estimated Cronbach’s alpha reliability coefficient was 0.750.

#### The Teacher’s Wellbeing Scale

The TWBS questionnaire has 16 items that measure three elements of wellbeing. The first element, workload wellbeing, pertains to problems related to workload and the following tensions. The second element, organizational wellbeing, pertains to educators’ understanding of the school as an enterprise consisting of understanding of school management and the culture regarding educators and education. The final element, learner interaction wellbeing, pertains to educators’ interactions with learners (understanding of learner conduct, motivation, and so on). The TWBS requests educators to score the degree to which various dimensions in their education impact their wellbeing. This method offers a related method for acquiring an insight into the central dimensions of education that impact educators. The items were recorded on a seven-point Likert-type scale ranging from 1 (Negatively) to 7 (Positively). In this study, the scale’s estimated Cronbach’s alpha reliability coefficient was 0.810.

### Data Collection Procedures

To meet the purposes of the study, by distributing questionnaires online *via* Wenjuanxing (an online questionnaire program to collect the data), data was successfully collected in the first 2 months of 2022. Altogether, 577 valid questionnaires were gleaned from different provinces and municipalities in China. To make it clear and understandable, the questionnaire is presented in Chinese. To increase the reliability and validity of the sample, participants were notified of how to properly fill in the questionnaire and give validated answers. They were also informed of their rights of withdrawal from the questionnaire if they sensed any discomfort in the process. There was no form of conflict between participants and researchers. Then, the collected data were double-checked for potential mistakes and limitations before being sent to the SPSS software for further analysis, which was, finally, conducted for the probe into the answers to research questions.

### Data Analysis

To answer the research questions of the study, after checking the necessary assumptions, a linear regression analysis was run.

## Results

First of all, the reliability of the three instruments was checked *via* running Cronbach’s Alpha. Table 1 demonstrates the reliability indices for the three questionnaires.

**Table 1:** The reliability indices for the three questionnaires.

	Mean		Std. Deviation	Variance	Cronbach’s Alpha
	Statistic	Std. Error	Statistic	Statistic	Index
Mindfulness	41.1404	0.38725	9.30200	86.527	0.720
Motivation	35.1248	0.23955	5.75408	33.109	0.750
Wellbeing	68.2964	0.66351	15.93805	254.021	0.810

As Table 1 indicates, the mindfulness, motivation, and wellbeing questionnaires all had reliability indices above 0.70 which are considered satisfactory (Hulin et al., 2001).

### Addressing the First Research Question

The first research question of the present study sought to explore if mindfulness significantly predicts wellbeing. To address this research question, a linear regression analysis was performed. This statistical test has a number of assumptions that need to be checked before running it. Initially, the sample size was checked. As Tabachnick and Fidell (2013, p. 123) contend, the sample size should equal “N > 50 + 8 m (where m = a number of independent variables).” In this study, the number of cases is 577 which is quite higher than the required sample size. Therefore, the first assumption was warranted. The next assumption was normality which was checked *via* consulting the Skewness and Kurtosis ratios. Table 2 displays the results of descriptive statistics for the mindfulness and wellbeing questionnaires.

**Table 2:** The results of descriptive statistics for the mindfulness and wellbeing questionnaires.

	N	Range	Minimum	Maximum	Mean		Std. Deviation	Variance	Skewness		Kurtosis	
	Statistic	Statistic	Statistic	Statistic	Statistic	Std. Error	Statistic	Statistic	Statistic	Std. Error	Statistic	Std. Error
Mindfulness	577	50.00	15.00	65.00	41.14	0.38	9.30	86.52	0.123	0.10	0.953	0.80
Wellbeing	577	88.00	24.00	112.00	68.29	0.66	15.93	254.0	0.110	0.10	1.368	0.80
Valid N (listwise)	577											

**Figure 1:**
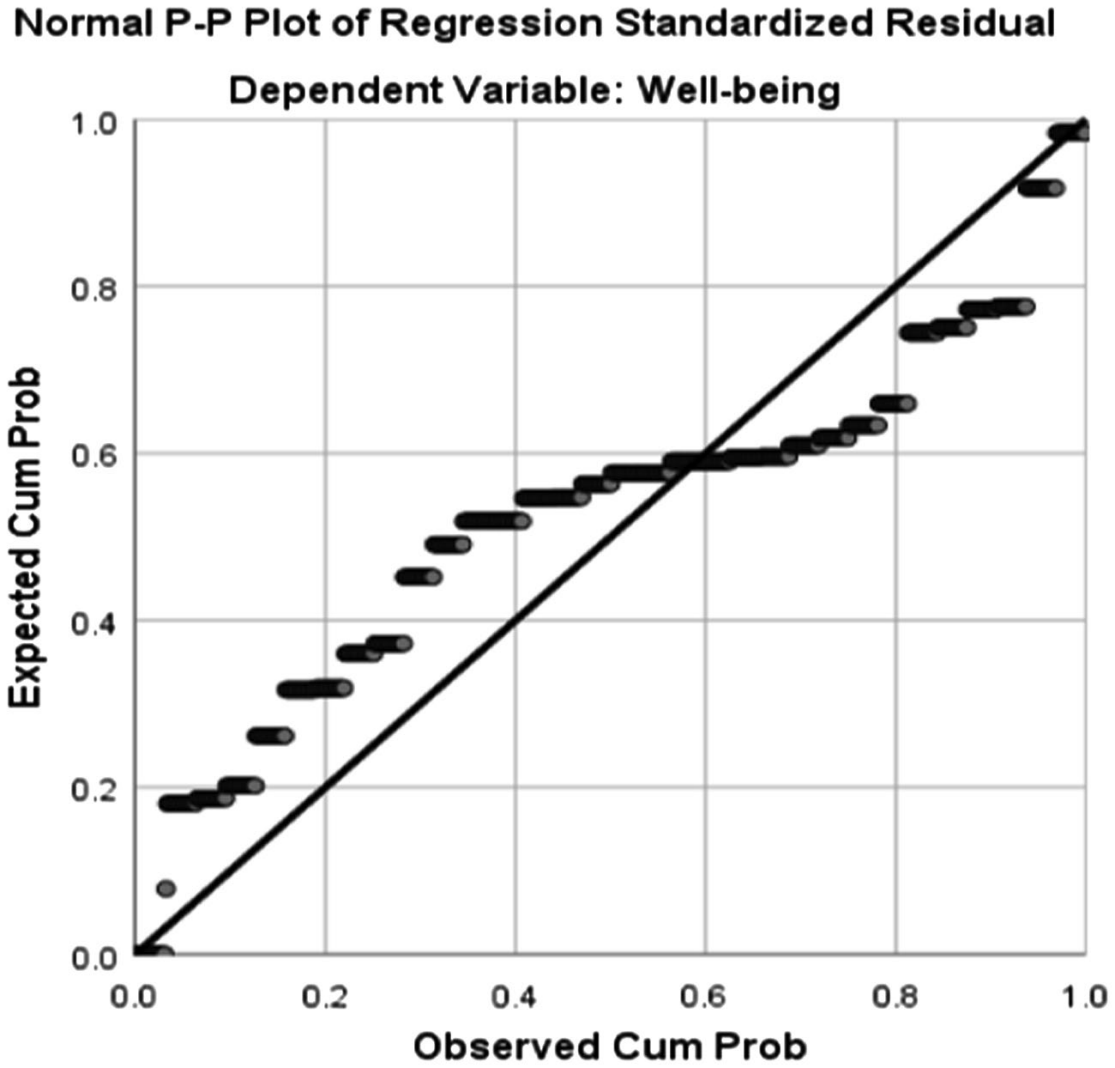
Normal probability plot (P–P) of the regression standardized residual (teachers’ wellbeing is the dependent variable).

**Figure 2:**
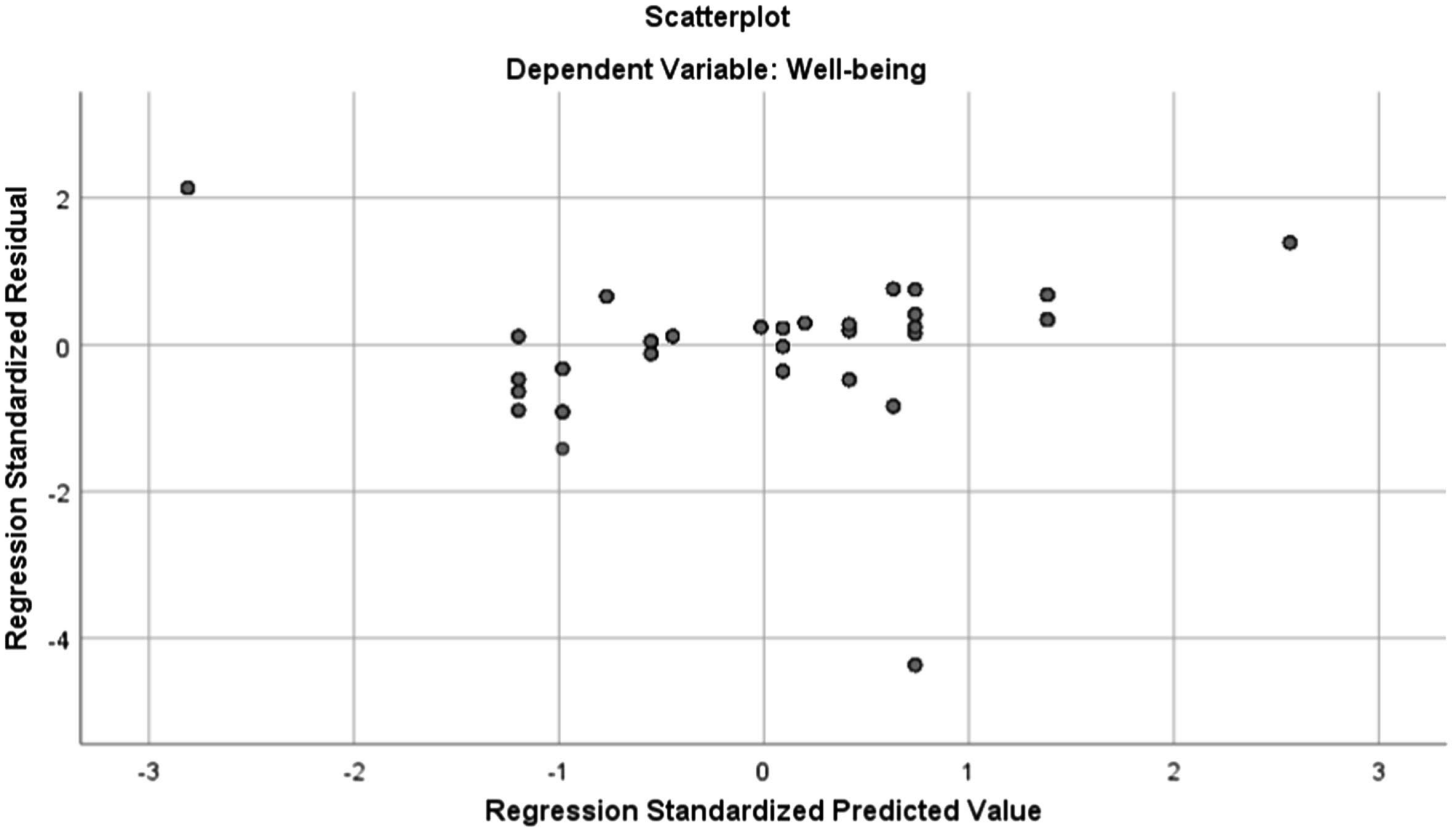
Scatter plot with teachers’ wellbeing as dependent variable.

As can be seen in Table 2, the Skewness and Kurtosis values fall within the range of +/−1.96 indicating that the two data sets are normally distributed. To check linearity, homoscedasticity, and independence of residuals assumptions the Normal Probability Plot (P–P) of the Regression Standardized Residual and the Scatterplot were inspected. Normal Probability Plot (P–P) of the Regression Standardized Residual and the Scatterplot are illustrated in Figures 1, 2, respectively.

As shown in the above scatterplot, the points are in a reasonably straight diagonal line from bottom left to top right which is an indication of no major deviations from normality and linearity conditions (Pallant, 2007).

As Figure 2 indicates, the points are spread in a rectangular shape which is an indication of no violation of linearity, homoscedasticity, and independence of residuals assumptions. Furthermore, no cases can be seen falling out of the range of +/− 3.3 suggesting the non-existence of outliers for the linear regression analysis. After checking the assumptions, the linear regression was run. Table 3 presents the model summary and ANOVA results for the linear regression analysis for exploring if mindfulness significantly predicts wellbeing.

**Table 3:** Model summary and ANOVA test of linear regression analysis for mindfulness as a predictor of wellbeing.

Model	R	*R* ^2^	Adjusted *R*^2^	Std. Error of the Estimate	*F*	df1	df2	Sig.
1	0.664^a^	0.441	0.440	11.92	453.92	1	575	0.000

a *Predictors: (Constant), Teachers’ Mindfulness. ^b^Dependent Variable: Teachers’ Wellbeing*.

As shown in Table 3, mindfulness explained about 44% of the variance in the dependent variable (Teachers’ wellbeing). Put it another way, teachers’ mindfulness made a 44% contribution to explaining the variance in teachers’ wellbeing. This amount of contribution was found significant as the *F* value was significant [*F* (1,575) =453.92, *p* = 0.00 < 0.05]. Thus, it can be inferred that mindfulness significantly predicts wellbeing.

### Addressing the Second Research Question

The second research question of this study set out to examine if mindfulness significantly predicts motivation. To address this research question, a linear regression analysis was carried out. The assumption of sample size was already checked and explained for research question one. The second assumption was normality which was checked *via* consulting the Skewness and Kurtosis ratios. Table 4 depicts the results of descriptive statistics for the mindfulness and motivation questionnaires.

**Table 4:** Descriptive statistics for the mindfulness and motivation questionnaires.

	N	Range	Minimum	Maximum	Mean		Std. Deviation	Variance	Skewness		Kurtosis	
	Statistic	Statistic	Statistic	Statistic	Statistic	Std. Error	Statistic	Statistic	Statistic	Std. Error	Statistic	Std. Error
Mindfulness	577	50.00	15.00	65.00	41.14	0.38	9.3020	86.52	0.123	0.10	0.953	0.80
Motivation	577	25.00	25.00	50.00	35.12	0.23	5.7540	33.10	0.120	0.10	0.808	0.80
Valid N (listwise)	577											

**Figure 3:**
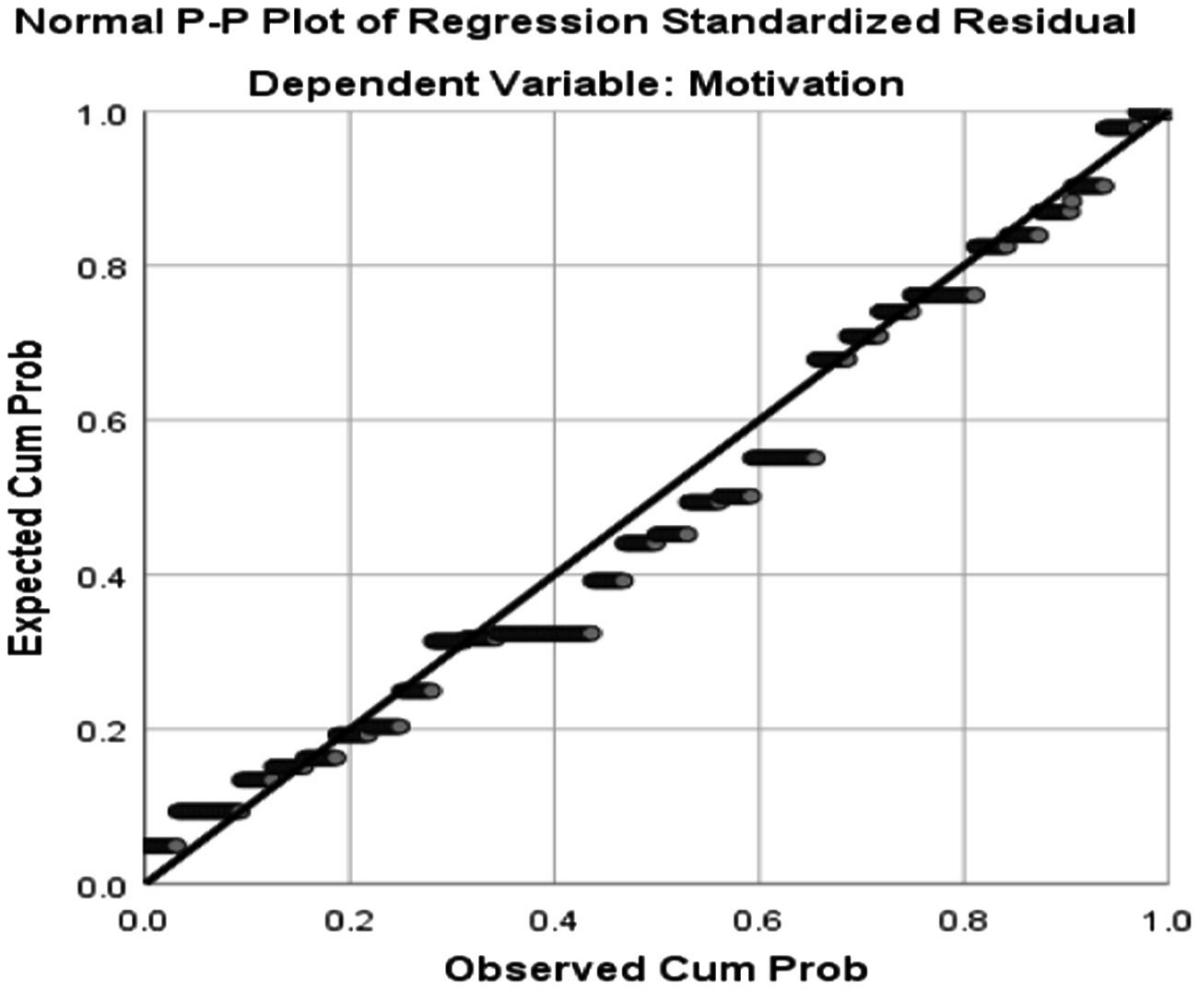
Normal probability plot (P–P) of the regression standardized residual (teachers’ motivation is the dependent variable).

**Figure 4:**
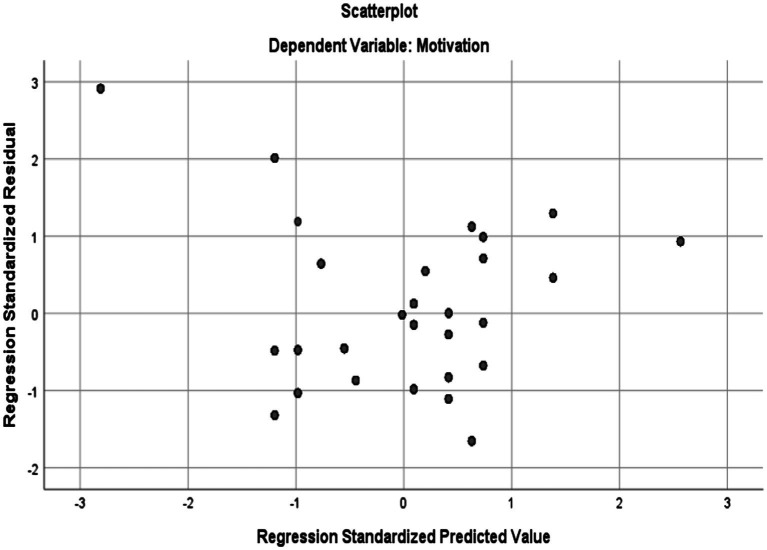
Scatter plot with teachers’ motivation as dependent variable.

Based on the information in Table 4, the Skewness and Kurtosis values are within the range of +/−1.96 indicating that the two data sets are normally distributed. To check linearity, homoscedasticity, and independence of residuals assumptions, the Normal Probability Plot (P–P) of the Regression Standardized Residual and the Scatterplot were consulted. Normal Probability Plot (P–P) of the Regression Standardized Residual and the Scatterplot are illustrated in Figures 3, 4, respectively.

As seen in the scatterplot above, the points are in a reasonably straight diagonal line from bottom left to top right indicating no major deviations from normality and linearity conditions (Pallant, 2007).

As seen in Figure 4, the points are scattered in a rectangular shape which is an indication of no violation of linearity, homoscedasticity, and independence of residuals assumptions. Furthermore, no cases can be seen lying out of the range of +/− 3.3 suggesting the non-existence of outliers for the linear regression analysis. After checking the assumptions, linear regression was performed. Table 5 displays the model summary and ANOVA results for the linear regression analysis for exploring if mindfulness significantly predicts motivation.

**Table 5:** Model summary and ANOVA test of linear regression analysis for mindfulness as a predictor of motivation.

Model	R	*R* ^2^	Adjusted *R*^2^	Std. Error of the Estimate	*F*	df1	df2	Sig.
1	0.780^a^	0.609	0.608	3.60	894.940	1	575	0.000^b^

a *Predictors: (Constant), Teachers’ Mindfulness. ^b^Dependent Variable: Teachers’ Motivation*.

As presented in Table 5, mindfulness explained about 60% of the variance in the dependent variable (Teachers’ Motivation). Put it differently, teachers’ mindfulness made a 60% contribution to explaining the variance in teachers’ motivation. This amount of contribution was found significant as the *F* value was significant [*F* (1,575) =894.94, *p* = 0.00 < 0.05]. Thus, it can be inferred that mindfulness significantly predicts motivation.

## Discussion

This study aimed at investigating if mindfulness significantly predicts wellbeing. Likewise, the study set out to investigate if mindfulness significantly predicts motivation. The results of linear regression analysis indicated that teachers’ mindfulness could significantly predict both teachers’ wellbeing and motivation. The self-determination theory (SDT) account proposes that one way through which mindfulness improves wellbeing is by enabling greater independent control of manner, thereby being related to a higher level of consistency and fewer problems when acting, greater fulfillment, and lower level of anxiety (Shannon et al., 2020). In other words, SDT forecasts that independence to a certain degree arbitrates the connections between mindfulness and wellbeing results. This is in line with results by Weinstein et al. (2009) that more mindful individuals both handle anxiety more successfully when they face it and have a lower level of anxiety by making more self-endorsed and well-combined decisions. Mindfulness coaching exercises are successful in enhancing educators’ mental health (like educators’ wellbeing) and lowering educators’ anxiety and burnout, and these influences have been mainly explored through individual first-person measures (Hue and Lau, 2015). Mindfulness is a method to start promoting connections and also improves teachers’ wellbeing which must always be a prime concern for schools to motivate and support.

The results are in line with Hwang et al. (2017) who considers teachers’ wellbeing and their presentation as the values of mindfulness training. Mental wellbeing assists educators with creating a constructive connection with their learners, which results in a greater educational presentation. Moreover, it is a beneficial concept that results in greater career fulfillment and elevated students’ presentation (Kidger et al., 2016). In a study conducted by Braun et al. (2019) who investigated educators’ current degrees of mindfulness regarding student interactions, educator career wellbeing, and subjective wellbeing, the results are consistent with the study of fifty-eight educators in the middle school. They showed that educators with higher degrees of mindfulness had decreased degrees of career tension, exhaustion, depression, and apprehension signs, and also had better degrees of observers’ scores of affective supportive interactions with learners in their most stressful classes. The results are consistent with previous research carried out by Hue and Lau (2015) who stated that the overall pattern of correlation suggests that mindfulness was in a positive relationship with wellbeing and a negative relationship with sadness, worry, and anxiety. The findings are consistent with former inquiries in which there was significant growth in wellbeing due to mindfulness intervention (Shapiro et al., 2008). The results support the study conducted by Jennings (2015) who found the relationship between mindfulness competence, educators’ career health and wellbeing, and class activities were in preschool educators.

The results are in agreement with Lomas et al. (2017) who concluded that mindfulness has a greatly constructive effect on teachers’ wellbeing and the outcomes portrayed constructive results for mindfulness, sympathy, empathy, emotive control, fulfillment, and career presentation. Furthermore, being involved in mindfulness meditation improves the skill of concentrating and increasing attention, which is a feasible method to bring about mental wellbeing (Wolkin, 2015). The current research suggests that enhancing mindfulness abilities is one prosperous method to support educators’ wellbeing (Kirby et al., 2017), and possibly enhance their degree of motivation regarding the fact that mindfulness is taken into account as a flexible element. The outcomes are following Chiesa et al. (2011) who explored the mindfulness and mental wellbeing of educators, and they demonstrated that mindfulness coaching elevated mental abilities and control techniques which result in elevated maintainability.

## Conclusion and Implications

The outcomes of such research, which explores educators’ mental characteristics and mindfulness, can increase the consciousness of students, educator trainers, policymakers, content creators, and other scholars in the domain. The results will assist with improving consciousness of the significance of educators’ wellbeing and motivation, thereby advancing helpful and constructive career experiences for educators. Mindfulness is proposed as a sort of expert enhancement to deal with the demands of education, and many applications are present that offer this specific teaching (Roeser et al., 2012). In line with the numerous studies on workplace mindfulness, results indicate that mindfulness is advantageous for educators; educators in such programs display enhancements in mindfulness competencies, career self-kindness, and wellbeing, and decreases in tension, depression, tension, and exhaustion (Jennings et al., 2013). It is believed that educators with higher mindfulness can recognize, adjust, and handle their cognitive and affective experiences such that have lower tension, depression, and anxiety. It is hypothesized that lower stress leads to newfound power that educators are able to employ to foster constructive relations with learners. The results in this research advocate these hypotheses, and consequently help the current work that aims at educator’s mindfulness as a flexible element in expert improvement programs for decreasing educator tension and enhancing educator–learner interactions within the class (Roeser et al., 2012; Jennings et al., 2013). The plans of mindfulness of classroom teachers are barely beginning to be explored, utilizing several studies to figure out the results of mindfulness exercises for teachers. There is still no point of view concerning the suggested framework of this kind of learning. Educators who exercise mindfulness themselves are more successful in instructing mindfulness and gaining themselves. Mindfulness programs in teacher education and educator coaching would enhance further execution of mindfulness at schools (Bailey et al., 2018). Because educators mostly have motivation issues, mindfulness is a proper treatment to raise their awareness about themselves. In this regard, it is stated that mindfulness aids educators remove their motivational problems in class and mindful educators can have higher enthusiasm and tendency to education. Based on the results of this research increasing mindfulness will decrease tension. Thus, teachers can be persuaded to learn mindfulness as an essential element for managing tension and burnout by letting them know the advantages of psychological awareness resulting in higher motivation.

Mindfulness is related to the motivation and mental and behavioral wellbeing of humans (Ryan and Deci, 2020). Based on their conclusion, with the aid of the present moment consciousness, individuals begin to discover their internal self and begin realizing their requirements and senses, which subsequently results in the individual’s higher connection to their true self, mind, and sensations. Such connection enables the individuals to recognize why they do what they do. Because mindfulness calls for higher awareness regarding the moment and thus greater attention regarding the current moment, it lets individuals become into their real selves and turn into what they really are (Weinstein et al., 2009). If such self-consciousness is taken into consideration in the working context, people can be instructed to make decisions consciously to have higher engagement in their activities about their work and consequently build more sense in their actions while they enhance their awareness.

Based on the research findings, mindfulness is one of the resources of educators’ wellbeing, and comprehending such elements affects educators’ wellbeing in the job environment that is critical as a reference for preparing educators’ psychological health improvement programs and building a framework for their expert progress. Because mindfulness lessens tension, it is considered to be a proactive method to assist in-carrier educators to improve function and wellbeing (McCallum et al., 2017). The contributions of comprehending educators’ wellbeing and motivation reach learners since these factors are connected to instructing effectiveness, flexibility in instruction, and learners’ motivation and success (Duckworth et al., 2009). Moreover, the immersion of mindfulness coaching in educator coaching programs has the possibility of improving psychological health, motivation, wellbeing, and anxiety reduction; thereby, lowering the possible risk of sadness and burnout among instructing experts. Furthermore, material developers creators can be profited from by taking into consideration the mindfulness of educators, resulting in a less anxious and defensive setting of education, greater motivation, and increased wellbeing. Thus, class pressure is eliminated, a calm class ambiance can be developed, and students’ motivation and engagement can be improved.

## Data Availability Statement

The original contributions presented in the study are included in the article/supplementary material, further inquiries can be directed to the corresponding author.

## Ethics Statement

The studies involving human participants were reviewed and approved by University of International Business and Economics Academic Ethics Committee. The patients/participants provided their written informed consent to participate in this study.

## Author Contributions

All authors listed have made a substantial, direct, and intellectual contribution to the work, and approved it for publication.

## Conflict of Interest

The authors declare that the research was conducted in the absence of any commercial or financial relationships that could be construed as a potential conflict of interest.

## Publisher’s Note

All claims expressed in this article are solely those of the authors and do not necessarily represent those of their affiliated organizations, or those of the publisher, the editors and the reviewers. Any product that may be evaluated in this article, or claim that may be made by its manufacturer, is not guaranteed or endorsed by the publisher.

## References

[ref1] Acton R. Glasgow P. (2015). Teacher well-being in neoliberal contexts: A review of the literature. Aus. J. Teach. Educ. 40, 40–63. doi: 10.14221/ajte.2015v40n8.6

[ref2] Altan S. Lane J. F. Dottin E. (2019). Using habits of mind, intelligent behaviors, and educational theories to create a conceptual framework for developing effective teaching dispositions. J. Teach. Educ. 70, 169–183. doi: 10.1177/0022487117736024

[ref3] Bailey N. Owen J. Chambers R. Hassed C. Jones A. Wootten A. (2018). Evidence based guidelines for mindfulness in schools: A guide for teachers and school leaders. Smiling Mind. Available at: https://static1.squarespace.com/static/5a2f40a41f318d38ccf0c819/t/5b28988170a6ad07781beeb9/1529387171804/smiling-mind-mindfulness-guidelines-for-schools-whitepaper (Accessed February 2022).

[ref5] Bernay R. (2014). Mindfulness and the beginning teacher. Aus. J. Teach. Educ. 39, 58–69. doi: 10.3316/informit.480460980498552

[ref6] Bishop S. R. Lau M. Shapiro S. Carlson L. Anderson N. Carmody J.. (2004). Mindfulness: A proposed operational definition. Clin. Psychol. Sci. Pract. 11, 230–241. doi: 10.1093/clipsy.bph077

[ref7] Braun S. S. Roeser R. W. Mashburn A. J. Skinner E. (2019). Middle school teachers' mindfulness, occupational health and well-being, and the quality of teacher-student interactions. Mindfulness 10, 245–255. doi: 10.1007/s12671-018-0968-2

[ref10] Brown M. Ralph S. Brember I. (2002). Change-linked work-related stress in British teachers. Res. Educ. 67, 1–12. doi: 10.7227/RIE.67.1

[ref8] Brown K. W. Ryan R. M. (2003). The benefits of being present: mindfulness and its role in psychological well-being. J. Pers. Soc. Psychol. 84, 822–848. doi: 10.1037/0022-3514.84.4.822, PMID: 12703651

[ref9] Brown K. W. Ryan R. M. Creswell J. D. (2007). Mindfulness: theoretical foundations and evidence for its salutary effects. Psychol. Inq. 18, 211–237. doi: 10.1080/10478400701598298

[ref11] Carmody J. Baer R. A. (2008). Relationships between mindfulness practice and levels of mindfulness, medical and psychological symptoms and well-being in a mindfulness based stress reduction program. J. Behav. Med. 31, 23–33. doi: 10.1007/s10865-007-9130-7, PMID: 17899351

[ref12] Chiesa A. Calati R. Serretti A. (2011). Does mindfulness training improve cognitive abilities? A systematic review of neuropsychological findings. Clin. Psychol. Rev. 31, 449–464. doi: 10.1016/j.cpr.2010.11.003, PMID: 21183265

[ref13] Collie R. J. Shapka J. D. Perry N. E. (2012). School climate and social-emotional learning: predicting teacher stress, job satisfaction, and teaching efficacy. J. Educ. Psychol. 104, 1189–1204. doi: 10.1037/a0029356

[ref001] Day C. Gu Q. (2014). Response to margolis, hodge and alexandrou: misrepresentations of teacher resilience and hope. J. Educ. Teach. 40, 409–412. doi: 10.1080/02607476.2014.948707

[ref14] Deci E. L. Ryan R. M. (2016). “Optimizing students’ motivation in the era of testing and pressure: A self-determination theory perspective,” in Building Autonomous Learners: Perspectives From Research and Practice Using Self-Determination Theory. eds. Liu W. C. Wang J. C. K. Ryan R. M. (Singapore: Springer Singapore), 9–29.

[ref15] Derakhshan A. (2021). The predictability of Turkman students’ academic engagement through Persian language teachers’ nonverbal immediacy and credibility. J. Teach. Persian Speakers Other Lang. 10, 3–26. doi: 10.30479/JTPSOL.2021.14654.1506

[ref17] Dörnyei Z. (2005). The Psychology of the Language Learner: Individual Differences in Second Language Acquisition. Mahwah, NJ: Lawrence Erlbaum.

[ref18] Dörnyei Z. Ushioda E. (2011). Teaching and Researching Motivation. Harlow: Pearson Education Limited.

[ref19] Duckworth A. L. Quinn P. D. Seligman M. E. P. (2009). Positive predictors of teacher effectiveness. J. Posit. Psychol. 4, 540–547. doi: 10.1080/17439760903157232

[ref20] Dundas I. Thorsheim T. Hjeltnes A. Binder P. E. (2016). Mindfulness based stress reduction for academic evaluation anxiety: a naturalistic longitudinal study. J. Coll. Stud. Psychother. 30, 114–131. doi: 10.1080/87568225.2016.1140988, PMID: 27227169 PMC4867855

[ref21] Dweik B. S. Awajan N. W. (2013). Factors that enhance English language teachers’ motivation in Jordanian secondary schools. Eng. Ling. Res. 2, 33–42. doi: 10.5430/elr.v2n1p33

[ref23] Fokkens-Bruinsma M. Canrinus E. T. Ten Hove M. Rietveld L. (2018). The relationship between teachers' work motivation and classroom goal orientation. Pedagogische Stu. 95, 86–100.

[ref24] Frank J. L. Jennings P. A. Greenberg M. T. (2016). Validation of the mindfulness in teaching scale. Mindfulness 7, 155–163. doi: 10.1007/s12671-015-0461-0

[ref25] Fredrickson B. L. (2001). The role of positive emotions in positive psychology: The broaden-and-build theory of positive emotions. Am. Psychol. 56, 218–226. doi: 10.1037/0003-066X.56.3.218 11315248 PMC3122271

[ref002] Gillet N. Berjot S. Vallerand R. J. Amoura S. Rosnet E. (2012). Examining the motivation performance relationship in competitive sport: a cluster-analytic approach. Int. J. Sport Psychol. 43, 79–102.

[ref26] Giorgi G. Shoss M. Di Fabio A. (2017). Editorial: from organizational welfare to business success: higher performance in healthy organizational environments. Front. Psychol. 8:720. doi: 10.3389/fpsyg.2017.00720, PMID: 28588519 PMC5440579

[ref27] Gold E. Smith A. Hopper I. Herne D. Tansey G. Hulland C. (2010). Mindfulness-based stress reduction (MBSR) for primary school teachers. J. Child Fam. Stud. 19, 184–189. doi: 10.1007/s10826-009-9344-0

[ref003] Gordon D. G. (2002). Discipline in the music classroom: One component contributing to teacher stress. Music Edu. Res. 4, 157–165. doi: 10.1080/14613800220119831

[ref28] Greenfield B. (2015). How can teacher resilience be protected and promoted? Educ. Child Psychol. 32, 53–68.

[ref29] Greenier V. Derakhshan A. Fathi J. (2021). Emotion regulation and psychological well-being in teacher work engagement: A case of British and Iranian English language teachers. System 97:102446. doi: 10.1016/j.system.2020.102446

[ref31] Hiver P. Dörnyei Z. (2017). Language teacher immunity: A double-edged sword. Appl. Linguis. 38:amv034. doi: 10.1093/applin/amv034

[ref32] Hue M. T. Lau N. S. (2015). Promoting well-being and preventing burnout in teacher education: A pilot study of a mindfulness-based program for pre-service teachers in Hong Kong. Teach. Dev. 19, 381–401. doi: 10.1080/13664530.2015.1049748

[ref33] Hulin C. Netemeyer R. Cudeck R. (2001). Can a reliability coefficient be too high? J. Consum. Psychol. 10, 55–69. doi: 10.1207/S15327663JCP1001and2_05

[ref34] Hwang M. H. Lim H. J. Ha H. S. (2017). Effects of grit on the academic success of adult female students at Korean open university. Psychol. Rep. 121, 705–725. doi: 10.1177/0033294117734834, PMID: 29298549

[ref35] Jennings P. A. (2015). Early childhood teachers’ well-being, mindfulness, and self-compassion in relation to classroom quality and attitudes towards challenging students. Mindfulness 6, 732–743. doi: 10.1007/s12671-014-0312-4

[ref37] Jennings P. A. Frank J. L. Snowberg K. E. Coccia M. A. Greenberg M. T. (2013). Improving classroom learning environments by cultivating awareness and resilience in education: results of a randomized controlled trial. Sch. Psychol. Q. 28, 374–390. doi: 10.1037/spq0000035, PMID: 24015983

[ref36] Jennings P. A. Greenberg M. T. (2009). The prosocial classroom: teacher social and emotional competence in relation to student and classroom outcomes. Rev. Educ. Res. 79, 491–525. doi: 10.3102/0034654308325693

[ref38] Kabat-Zinn J. (2003). Mindfulness-based interventions in context: past, present, and future. Clin. Psychol. Sci. Pract. 10, 144–156. doi: 10.1093/clipsy/bpg016

[ref39] Kern M. L. Benson L. Steinberg E. A. Steinberg L. (2016). The EPOCH measure of adolescent well-being. Psychol. Assess. 28, 586–597. doi: 10.1037/pas0000201, PMID: 26302102

[ref40] Kidger J. Brockman R. Tilling K. Campbell R. Ford T. Araya R.. (2016). Teachers' well-being and depressive symptoms, and associated risk factors: A large cross-sectional study in English secondary schools. J. Affect. Disord. 192, 76–82. doi: 10.1016/j.jad.2015.11.054, PMID: 26707351

[ref41] Kim-Prieto C. Diener E. Tamir M. Scollon C. Diener M. (2005). Integrating the diverse definitions of happiness: A time-sequential framework of subjective well-being. J. Happiness Stud. 6, 261–300. doi: 10.1007/s10902-005-7226-8

[ref42] Kirby J. N. Tellegen C. L. Steindl S. R. (2017). A meta-analysis of compassion-based interventions: current state of knowledge and future directions. Behav. Ther. 48, 778–792. doi: 10.1016/j.beth.2017.06.003, PMID: 29029675

[ref43] Komarraju M. Karau S. J. Schmeck R. R. (2009). Role of the big five personality traits in predicting college students' academic motivation and achievement. Learn. Individ. Differ. 19, 47–52. doi: 10.1016/j.lindif.2008.07.001

[ref44] Leary M. R. Tate E. B. (2007). The multi-faceted nature of mindfulness. Psychol. Inq. 18, 251–255. doi: 10.1080/10478400701598355

[ref45] Lomas T. Medina J. C. Ivtzan I. Rupprecht S. Eiroa-Orosa F. J. (2017). The impact of mindfulness on the well-being and performance of educators: A systematic review of the empirical literature. Teach. Teach. Educ. 61, 132–141. doi: 10.1016/j.tate.2016.10.008

[ref46] Lyubomirsky S. King L. Diener E. (2005). The benefits of frequent positive affect: does happiness authenticated? Psychol. Bull. 131, 803–855. doi: 10.1037/0033-2909.131.6.803, PMID: 16351326

[ref47] MacIntyre P. D. Dewaele J. M. Macmillan N. Li C. (2019). “The emotional underpinnings of Gardner’s attitudes and motivation test battery,” in Contemporary Language Motivation Theory. eds. MacIntyre P. D. Al-Hoorie A. (Bristol: Multilingual Matters), 57–79.

[ref48] MacIntyre P. Gregersen T. (2012). Emotions that facilitate language learning: The positive-broadening power of the imagination. Stu. Sec. Lang. Learning Teach. 2, 193–213. doi: 10.14746/ssllt.2012.2.2.4

[ref49] Martinez N. Sinedino L. D. P. Bisinotto R. S. Daetz R. Risco C. A. Galvão K. N.. (2016). Effects of oral calcium supplementation on productive and reproductive performance in Holstein cows. J. Dairy Sci. 99, 8417–8430. doi: 10.3168/jds.2015-10529, PMID: 27423945

[ref50] McCallum F. Price D. Graham A. Morrison A. (2017). Teacher well-being: A review of the literature. AIS: NSW, the University of Adelaide, Australia. Available at: https://www.aisnsw.edu.au/Educationalresearch/documents/commissionedResearch/Teacherwell-beingAreviewoftheliterature-FayeMcCallumAISNSW2017 (Accessed February 2022).

[ref51] McCullough M. Van Oyen Witvliet C. (2005). “The psychology of forgiveness,” in Hanbook of Positive Psychology. eds. Snyder C. R. Lopez S. J. (Oxford: Oxford University Press), 34–56.

[ref52] McIntyre T. M. McIntyre S. E. Francis D. J. (2017). “Educator stress: An occupational health perspective. In current knowledge on the nature, prevalence, sources and potential impact of teacher stress,” in Implications of an Occupational Health Perspective for Educator Stress Research. eds. McIntyre T. M. McIntyre S. E. Francis D. J. (Cham, Switzerland: Springer), 485–505.

[ref53] Mercer S. (2018). Psychology for language learning: spare a thought for the teacher. Lang. Teach. 51, 504–525. doi: 10.1017/S0261444817000258

[ref54] Mercer S. Gregersen T. (2020). Teacher Well-Being. Oxford: Oxford University Press.

[ref55] Mercer S. Oberdorfer P. Saleem M. (2016). “Helping language teachers to thrive: using positive psychology to promote teachers’ professional well-being,” in Positive Psychology Perspectives on Foreign Language Learning and Teaching. eds. Gabryś-Barker D. Gałajda D. (Switzerland: Springer), 213–229.

[ref56] Neves de Jesus S. Lens W. (2005). An integrated model for the study of teacher motivation. Appl. Psychol. 54, 119–134. doi: 10.1111/j.1464-0597.2005.00199.x

[ref57] O’Driscoll M. Byrne S. McGillicuddy A. Lambert S. Sahm L. (2018). The effects of mindfulness-based interventions for health and social care undergraduate students. Psychol. Health Med. 22, 851–865. 10.1080/13548506.2017.1280178 28103700

[ref58] Pallant J. (2007). SPSS Survival Manual. 4th *Edn*. United States: McGraw-Hill Education.

[ref59] Roeser R. W. Skinner E. Beers J. Jennings P. A. (2012). Mindfulness training and teachers’ professional development: An emerging area of research and practice. Child Dev. Perspect. 6, 167–173. doi: 10.1111/j.1750-8606.2012.00238.x

[ref60] Ryan R. M. Deci E. L. (2020). Intrinsic and extrinsic motivation from a self-determination theory perspective: definitions, theory, practices, and future directions. Contemp. Educ. Psychol. 61:101860. doi: 10.1016/j.cedpsych.2020

[ref61] Schoeberlein D. Sheth S. (2009). Mindful Teaching and Teaching Mindfulness: A Guide for Anyone Who Teaches Anything. Somerville, United States: Wisdom Publications.

[ref62] Schussler D. L. Jennings P. A. Sharp J. E. Frank J. L. (2016). Improving teacher awareness and well-being through CARE: a qualitative analysis of the underlying mechanisms. Mindfulness 7, 130–142. doi: 10.1007/s12671-015-0422-7

[ref63] Seligman M. E. P. (2011). Flourish: A Visionary New Understanding of Happiness and Well-Being. New York: Free Press.

[ref64] Shannon S. Hanna D. Leavey G. Haughey T. Neill D. Breslin G. (2020). The association between mindfulness and mental health outcomes in athletes: testing the mediating role of autonomy satisfaction as a core psychological need. Int. J. Sport Exer. Psychol. 1–16. doi: 10.1080/1612197X.2020.1717578

[ref65] Shapiro S. L. Oman D. Thoresen C. E. Plante T. G. Flinders T. (2008). Cultivating mindfulness: effects on well-being. J. Clin. Psychol. 64, 840–862. doi: 10.1002/jclp.20491 18484600

[ref66] Sharp J. E. Jennings P. A. (2016). Strengthening teacher presence through mindfulness: what educators say about the cultivating awareness and resilience in education program. Mindfulness 7, 209–218. doi: 10.1007/s12671-015-0474-8

[ref67] Singh K. (2011). Study of achievement motivation in relation to academic achievement of students. Int. J. Educ. Plan. Admin. 1, 161–171.

[ref68] Song X. He X. (2021). Teachers’ dispositions toward mindfulness in EFL/ESL classrooms in teacher student interpersonal relationships. Front. Psychol. 12:754998. doi: 10.3389/fpsyg.2021.754998, PMID: 34603174 PMC8481581

[ref69] Tabachnick B. G. Fidell L. S. (2013). Using Multivariate Statistics. 6th *Edn*. Boston: Pearson Education.

[ref70] Taylor C. Harrison J. Haimovitz K. Oberle E. Thomson K. Schonert-Reich K.. (2016). Examining ways that a mindfulness-based intervention reduces stress in public school teachers: A mixed-methods study. Mindfulness 7, 115–129. doi: 10.1007/s12671-015-0425-4

[ref71] Ushioda E. (2008). “Motivation and good language learners,” in Lessons From Good Language Learners. ed. Griffiths C. (Cambridge, UK: Cambridge University Press), 19–34.

[ref72] Wang Y. L. Derakhshan A. Zhang L. J. (2021). Researching and practicing positive psychology in second/foreign language learning and teaching: The past, current status and future directions. Front. Psychol. 12:731721. doi: 10.3389/fpsyg.2021.731721, PMID: 34489835 PMC8417049

[ref73] Wang Y. L. Guan H. F. (2020). Exploring demotivation factors of Chinese learners of English as a foreign language based on positive psychology. Rev. Arg. Clin. Psicol. 29, 851–861. doi: 10.24205/03276716.2020.116

[ref74] Wang Y. Liu C. (2016). Cultivate mindfulness: A case study of mindful learning in an English as a foreign language classroom. IAFOR J. Educ. 4, 141–155. doi: 10.22492/ije.4.2.08

[ref75] Wax R. (2016). A Mindfulness Guide for the Frazzled. Penguin Life, United Kingdom.

[ref76] Weinstein N. Brown K. W. Ryan R. M. (2009). A multi-method examination of the effects of mindfulness on stress attribution, coping, and emotional well-being. J. Res. Pers. 43, 374–385. doi: 10.1016/j.jrp.2008.12.008

[ref77] Wolkin J. (2015). How mindfulness Impacts well-being. Available at: https://www.mindful.org/how-mindfulness-impacts-well-being/ (February 2022).

[ref78] Yang J. (2021). The predictive role of Chinese EFL teachers’ individual self-efficacy and collective efficacy in their work engagement. Front. Psychol. 12:752041. doi: 10.3389/fpsyg.2021.752041, PMID: 34603168 PMC8481583

[ref79] Yuan R. Lee I. Xu H. Zhang H. (2020). The alchemy of teacher mindfulness: voices from veteran language teachers in China. Prof. Dev. Educ. 46, 1–17. doi: 10.1080/19415257.2020.1814383

